# Visual health and prevalence of dry eye syndrome among university students in Iraq and Jordan

**DOI:** 10.1186/s12886-022-02485-w

**Published:** 2022-06-14

**Authors:** Dina M. Abdulmannan, Abdallah Y. Naser, Omar khaleel Ibrahim, Abdullah Shakir Mahmood, Jamal Alyoussef Alkrad, Kanar Sweiss, Hamzeh Mohammad Alrawashdeh, Angga Prawira Kautsar

**Affiliations:** 1 College of Medicine, Umm Alqura University, Makkah, Saudi Arabia; 2 Department of Applied Pharmaceutical Sciences and Clinical Pharmacy, Faculty of Pharmacy, Isra University, Amman, Jordan; 3 College of Medicine, Ninevah University, Mosul, Iraq; 4 Department of Basic Pharmaceutical Sciences, Faculty of Pharmacy, Isra University, Amman, Jordan; 5 Department of Ophthalmology, Sharif Eye Centers, Irbid, Jordan; 6 Department of Health Sciences, Unit of Global Health, University of Groningen/University Medical Center Groningen, Groningen, the Netherlands; 7 Department of Pharmaceutics and Pharmaceutical Technology, Faculty of Pharmacy, Universitas Padjadjaran, Bandung, West Java Indonesia; 8 Center of Excellence in Higher Education for Pharmaceutical Care Innovation, Universitas Padjadjaran, Bandung, West Java Indonesia

**Keywords:** Dry eye syndrome, Iraq, Jordan, Students, Visual health

## Abstract

Dry eye syndrome (DES), is a multifactorial disease that affects the ocular surface and contributes to the ocular symptoms. The COVID-19 pandemic influenced the general population and university students' health in different ways. The pandemic forced many people including university students around the world to use virtual platforms on their digital devices, such as computers and smartphones, to work from a distance. This study aimed to explore the visual health and prevalence of dry eye syndrome among university students in Iraq and Jordan. This was a cross-sectional study that was conducted in Iraq and Jordan using online questionnaire tool for the duration between November 2021 and January 2022. University students in Jordan and Iraq were invited to participate in this study and formed the study population. No restrictions on study level or field of study were applied. A previously developed and validated questionnaire tools were used in this study (National Eye Institute Visual Functioning Questionnaire – 25 (VFQ-25) and the Women’s Health Study Questionnaire (WHS), which was developed by Schaumberg et al.). A total of 1,431 university students were involved in this study (1,018 students from Iraq, 71.1%). Around one third the study participants (29.0%) reported that have been diagnosed by a clinician as having dry eye syndrome. Around15.3% of the total study participants reported that they feel their eyes are dry (not wet enough) and 17.3% reported that they feel their eyes are irritated. Based on Women’s Health Study Questionnaire (WHS) criteria, a total of 479 participants (33.4%) are symptomatically diagnosed with DES. Students aged 27–29 years, those at their fifth year of study, and those who wear contact lenses are at higher risk of developing DYS compared to others. Dry eye syndrome is common health problem among university students. Further studies are required to identify other risk factors associated with DES. Future research should focus on identifying strategies that could help reduce the risk of developing DES as a result of the inevitability of long-term use of digital devices among many categories of society, including university students.

## Introduction

The thickness of the tear film is around 2 to 5 µm and it is found over the cornea. The tear film is composed of three different components, such as lipid, aqueous, and mucin [1]. Dry eye disease (DED), also known as dry eye syndrome (DES), is a multifactorial disease that affects the ocular surface and contributes to the ocular symptoms. The tear film loses its homeostasis and increases its osmolarity. DES not only affect the ocular surface, but also the lacrimal gland and meibomian gland [1].

Systemic medication, topical medication, skin disease, ophthalmic surgery, environmental factors such as pollution, low humidity, genetic factors, nutritional deficiency such as vitamin A deficiency, and cigarette smoking are all factors that contribute to DES [1, 2]. Furthermore, devices such as computers and mobile phones may reduce the blinking of the eyes while working on them [1, 3]. The global prevalence of DES varies from 5 to 50% [3]. There are 16 million confirmed DES diagnoses in the United States of America, and another 6 million people who have the symptoms but have not been diagnosed [4]. DES is most common in women compared to men, and it increases with age [3, 5–7]. Moreover, DES is more common in the Asian population compared to Europe and America [3].

Some DES symptoms are internal symptoms related to ametropia, accommodation or vergence problems (including ache, strain, and headache behind the eyes) [8], while others are external syndromes such as burning sensations in the eyes, feeling a sandy or foreign material inside the eyes, redness, blurry vision, pain in both the neck and shoulder, photophobia, tearing, focusing problems, and dryness [1, 8]. DES nowadays is linked to digital and computer devices, as DES increased 50% or more among device users. Most of the time, devices will cause ocular vision stress and cause dry eyes [8]. Devices are used by people of all ages. One European study found that more than 68% of children aged 3 and over are using computers [9]. Another study revealed that US and UK adults between the ages of 30 and 49 spend around 5 h daily on their mobiles [8].

The COVID-19 pandemic influenced the general population and university students’ health in different ways, including their social, mental, and clinical outcomes [10–17]. The pandemic forced many people around the world to use virtual platforms on their digital devices, such as computers and smartphones, to work from a distance. Young students also used them to study. All these precaution measurements increased the average screen time used to an alarming level. Not only does prolonged time on screens increase the suffering of DES [18], but also wearing facial masks contributes to the syndrome [19]. Wearing a facial mask resulted in exhaled air moving to the cornea and accelerating DES [19].

To diagnose DES, Tear Film, and Ocular Surface Society Dry Eye Workshop propose using either the Dry Eye Questionnaire (DEQ-5), Ocular Surface Disease Index (OSDI), or Symptom Assessment in Dry Eye (SANDE), followed by a non-invasive clinical test [20]. For mild to moderate symptoms, patients can use over-the-counter medication such as artificial tears eye drops; however, in more severe cases, patients should seek medical attention to determine the cause of the disease and treat it [21].

DES has a direct and indirect burden on the patients. It will affect their normal daily activities, their quality of life, their visual function, and their productivity at work. They will also be absent from work. It will lead to having to visit doctors and use medications [3, 22, 23]. To the best of our knowledge, there are limited studies that explored the prevalence of DES among university students in the Middle East and in Jordan and Iraq specifically. This study aimed to explore the visual health and prevalence of dry eye syndrome among university students in Iraq and Jordan.

## Method

### Study design

This was a cross-sectional study that was conducted in Iraq and Jordan using online questionnaire tool for the duration between November 2021 and January 2022. Qualtrics survey software was used to collect the participants’ responses.

### Study population

University students in Jordan and Iraq were invited to participate in this study and formed the study population. No restrictions on study level or field of study were applied.

### Sampling procedure

In order to recruit study participants, this study used a convenience sampling procedure. The URL to the survey was shared on social media channels (WhatsApp, Facebook, and Snapchat). The invitation letter was included in the questionnaire's cover letter. It gave a clear picture of the study’s goal and aims. Furthermore, the inclusion criteria were stated clearly. Participants were told that by completing the study questionnaire, they were giving their informed consent to participate in the study.

### Questionnaire tool

A previously developed and validated questionnaire tools were used in this study (National Eye Institute Visual Functioning Questionnaire – 25 (VFQ-25) [24] and the Women’s Health Study Questionnaire (WHS), which was developed by Schaumberg et al. [25]).

#### National eye institute visual functioning questionnaire – 25 (VFQ-25)

The NEI VFQ-25 is a 25-item questionnaire developed to evaluate vision impairment and health-related quality of life [26]. General vision, ocular pain, color vision, near activities, distant activities, social function, mental health, role issues, dependency, driving, and peripheral vision are among the 11 visual functioning subscales in the questionnaire [27]. The NEI VFQ-25 has six question types (global rating of health, difficulty, frequency, severity, agreement, and numeric) with different response categories (numeric with no labels, always–never, definitely true–definitely false, none–very severe, excellent–completely blind, and no difficulty at all–stopped doing this because of your eyesight) [28].

#### The women’s health study questionnaire

This questionnaire tool was developed by Schaumberg et al. [25]. This survey consisted of three questions: (1) Have you ever been diagnosed with dry eye syndrome by a clinician? (2) Do your eyes feel dry (not moist enough) on a regular basis? (3) How frequently do your eyes upset you? “Always,” “often,” “occasionally,” or “never” were all possible responses to the two questions about symptoms. The presence of either a previous clinical diagnosis of DES or severe symptoms of both dryness and irritation were used to identify DES according to WHS criteria (either constantly or often).

### Questionnaire translation

The first version of the questionnaire was written in English. We used a forward and backward translation technique to convert the original questionnaire into Arabic. Experts in the field of ophthalmology reviewed the translated version for face validity.

### Statistical analysis

The data for this study was analyzed using Statistical Packages for Social Sciences version 27 (SPSS Inc., Chicago, IL, US). The frequency and percentage of categorical data were displayed. The risk factors for developing DES were determined using binary logistic regression analysis. The statistical significance of the results was represented by a confidence interval (CI) of 95% (p < 0.05), and the level of significance was set at 5%.

## Results

### Demographic characteristics of study participants

Table 1 below describes the demographic characteristics of study participants. A total of 1,431 university students were involved in this study (1,018 students from Iraq, 71.1%). More than half of them (62.8%) are aged 18–23 years. Around half of them (52.5%) were studying subjects related to the medical field. Most of the study participants (79.0%) were single. A total of 53.7% were at years one to three of their program. Around 70% of the study participants reported that their monthly income is less than 700$. Around 8.0% of the study participants reported that they suffer from chronic conditions, and 13.1% reported that they use contact lenses. More than half of them (60.5%) reported that their screen time is more than 6 h per day.

**Table 1:** Demographic characteristics of study participants

**Demographic variable**	**Overall** (*n* = 1,431)	**Iraq** (*n* = 1,018)	**Jordan** (*n* = 413)
	Frequency (%)	Frequency (%)	Frequency (%)
**Gender**			
Females	999 (69.8%)	739 (72.6%)	260 (63.0%)
**Age category**			
18–20 years	389 (27.2%)	312 (30.6%)	77 (18.6%)
21–23 years	510 (35.6%)	378 (37.1%)	132 (32.0%)
24–26 years	296 (20.7%)	201 (19.7%)	95 (23.0%)
27–29 years	129 (9.0%)	79 (7.8%)	50 (12.1%)
30 years and above	107 (7.5%)	48 (4.7%)	59 (14.3%)
**Field of study**			
Medical college	751 (52.5%)	508 (49.9%)	243 (58.8%)
**Marital status**			
Single	1131 (79.0%)	823 (80.8%)	308 (74.6%)
Married	274 (19.1%)	181 (17.8%)	93 (22.5%)
Divorced	23 (1.6%)	12 (1.2%)	11 (2.7%)
Widowed	3 (0.2%)	2 (0.2%)	1 (0.2%)
**Year of study**			
First year	231 (16.1%)	196 (19.3%)	35 (8.5%)
Second year	291 (20.3%)	238 (23.4%)	53 (12.8%)
Third year	248 (17.3%)	197 (19.4%)	51 (12.3%)
Fourth year	308 (21.5%)	204 (20.0%)	104 (25.2%)
Fifth year	102 (7.1%)	24 (2.4%)	78 (18.9%)
Sixth year	23 (1.6%)	16 (1.6%)	7 (1.7%)
Higher education	228 (15.9%)	143 (14.0%)	85 (20.6%)
**Income level**			
Less than 700 USD	982 (68.6%)	760 (74.7%)	222 (53.8%)
700 – 1500 USD	302 (21.1%)	179 (17.6%)	123 (29.8%)
1500 – 2000 USD	91 (6.4%)	58 (5.7%)	33 (8.0%)
More than 2000 USD	56 (3.9%)	21 (2.1%)	35 (8.5%)
**Do you suffer from any chronic conditions?**			
Yes	107 (7.5%)	80 (7.9%)	27 (6.5%)
**Contact lens user**			
Yes	188 (13.1%)	142 (13.9%)	46 (11.1%)
**Screen time** (mobile, television, laptop, etc.…) **(hours/day)**			
Less than 2 h	40 (2.8%)	30 (2.9%)	10 (2.4%)
2–4 h	155 (10.8%)	116 (11.4%)	39 (9.4%)
4–6 h	370 (25.9%)	240 (23.6%)	130 (31.5%)
6–8 h	396 (27.7%)	292 (28.7%)	104 (25.2%)
8 h and above	470 (32.8%)	340 (33.4%)	130 (31.5%)

### Eye health profile

#### General health and vision

Around one quarter the study participants (23.6%) evaluated their overall health as very good and excellent. More than half of them (59.3%) reported that their eyesight using both eyes (with glasses or contact lenses, if wearing them) as very good and excellent, Table 2. A total of 16.7% of the study participants reported that they worry about their eyesight almost all the time (most of the time to all of the time). A total of 9.2% of them reported suffering from severe to very severe pain or discomfort in and around their eyes (for example, burning, itching, or aching), Table 2.

**Table 2:** Visual functioning – general health and vision and difficulty with activities

Item	Excellent		Very good			Good		Fair		Poor			
**General health and vision**													
**In general, would you say your overall health is:**	5.7%		17.9%			34.6%		35.6%		6.2%			
**At the present time, would you say your eyesight using both eyes (with glasses or contact lenses, if you wear them) is**	20.3%		39.0%			28.0%		5.9%		6.7%			
	**None of the time**		**A little of the time**			**Some of the time**		**Most of the time**		**All of the time**			
**How much of the time do you worry about your eyesight?**	23.0%		31.6%			28.7%		12.6%		4.1%			
	**None**		**Mild**			**Moderate**		**Severe**		**Very severe**			
**How much pain or discomfort have you had in and around your eyes (for example, burning, itching, or aching)? Would you say it is:**	17.0%		43.5%			30.3%		7.6%		1.6%			
**Difficulty with activities**													
	**No difficulty at all**		**A little difficulty**			**Moderate difficulty**		**Extreme difficulty**		**Stopped doing this because of your eyesight**			**Stopped doing this for other reasons or not interested in doing this**
**How much difficulty do you have reading ordinary print in newspapers? Would you say you have:**	61.4%		21.9%			11.0%		2.2%		0.9%			2.6%
**How much difficulty do you have doing work or hobbies that require you to see well up close, such as cooking, sewing, fixing things around the house, or using hand tools? Would you say:**	62.0%		22.0%			10.6%		3.4%		0.6%			1.5%
**Because of your eyesight, how much difficulty do you have finding something on a crowded shelf?**	60.6%		23.5%			10.1%		4.3%		0.8%			0.6%
**How much difficulty do you have reading street signs or the names of stores?**	52.8%		23.4%			12.6%		8.6%		2.1%			0.6%
**Because of your eyesight, how much difficulty do you have going down steps, stairs, or curbs in dim light or at night?**	67.6%		19.8%			8.7%		2.4%		0.9%			0.5%
**Because of your eyesight, how much difficulty do you have noticing objects off to the side while you are walking along?**	63.9%		21.0%			9.8%		3.7%		0.9%			0.8%
**Because of your eyesight, how much difficulty do you have seeing how people react to things you say?**	73.5%		15.0%			6.7%		3.4%		0.4%			1.0%
**Because of your eyesight, how much difficulty do you have picking out and matching your own clothes?**	81.3%		11.1%			4.5%		1.8%		0.4%			0.8%
**Because of your eyesight, how much difficulty do you have visiting with people in their homes, at parties, or in restaurants?**	83.3%		7.7%			4.8%		2.4%		0.6%			1.3%
**Because of your eyesight, how much difficulty do you have going out to see movies, plays, or sports events?**	72.6%		13.7%			6.6%		3.2%		1.2%			2.7%
**Are you currently driving, at least once in a while?**													
Yes	44.0%												
**If your answer was no for the previous question, have you never driven a car or have you given up driving?**													
Never drove	90.5%												
Gave up	9.5%												
**IF YOU GAVE UP DRIVING: Was that mainly because of your eyesight, mainly for some other reason, or because of both your eyesight and other reasons?**													
Mainly eyesight	4.7%												
Mainly other reasons	85.9%												
Both eyesight and other reasons	9.4%												
	**No difficulty at all**			**A little difficulty**			**Moderate difficulty**				**Extreme difficulty**		
**IF CURRENTLY DRIVING: How much difficulty do you have driving during the daytime in familiar places? Would you say you have:**	76.8%			18.3%			4.3%				0.6%		
	**No difficulty at all**	**A little difficulty**			**Moderate difficulty**		**Extreme difficulty**		**Stopped doing this because of your eyesight**			**Stopped doing this for other reasons or not interested in doing this**	
**How much difficulty do you have driving at night? Would you say you have:**	50.7%	29.8%			11.7%		5.7%		0.4%			1.6%	
**How much difficulty do you have driving in difficult conditions, such as in bad weather, during rush hour, on the freeway, or in city traffic? Would you say you have:**	42.5%	36.8%			11.9%		5.1%		1.0%			2.7%	

#### Difficulty with activities

When the participants were asked about difficulty with activities that they face related to their visual functioning, the degree of difficulty for different activities ranged between 16.7% to 47.2%. The most commonly reported activities for which the participants reported to face difficulties were reading street signs or the names of stores, reading ordinary print in newspapers, and finding something on a crowded shelf were 47.2%, 39.4%, and 38.6% of the participants reported some sort of difficulties while performing these activities, respectively, Table 2.

Less than half of the study participants (44.0%) reported that they are currently driving, at least once in a while. For those who reported that they do not currently drive and gave up, 4.7% of them reported that this was mainly because of their eyesight. For those who currently driving, 23.2% reported some sort of difficulty driving during the daytime in familiar places, Table 2. Around half of the study participants (49.3%) reported having some sort of difficulty driving at night and 47.5% reported some sort of difficulty driving in difficult conditions, such as in bad weather, during rush hour, on the freeway, or in city traffic, Table 2.

#### Responses to vision problems

Around half of the study participants (50.8%) reported that at some point of the time they accomplish less than what they would like because of their vision. Similar percentage of them (47.2%) reported that they accomplish less than what they would like because of their vision, and 64.0% of them reported that pain or discomfort in or around their eyes, for example, burning, itching, or aching, keep them from doing what they’d like to be doing, Table 3.

**Table 3:** Visual functioning—responses to vision problems

Responses to vision problems					
	**None of the time**	**A little of the time**	**Some of the time**	**Most of the time**	**All of the time**
**Read categories**					
Do you accomplish less than you would like because of your vision?	49.2%	15.9%	12.2%	11.9%	10.8%
Are you accomplish less than you would like because of your vision?	52.8%	17.3%	11.1%	8.7%	10.1%
How much does pain or discomfort in or around your eyes, for example, burning, itching, or aching, keep you from doing what you’d like to be doing? Would you say:	36.0%	27.8%	19.2%	10.4%	6.6%
	**Definitely false**	**Mostly false**	**Not sure**	**Mostly true**	**Definitely true**
I stay home most of the time because of my eyesight	67.8%	14.6%	5.6%	3.9%	8.1%
I feel frustrated a lot of the time because of my eyesight	58.5%	13.6%	8.7%	10.1%	9.2%
I have much less control over what I do, because of my eyesight	56.5%	15.4%	10.7%	9.0%	8.4%
Because of my eyesight, I have to rely too much on what other people tell me	65.7%	12.9%	7.3%	7.2%	6.9%
I need a lot of help from others because of my eyesight	69.1%	12.6%	6.2%	5.8%	6.3%
I worry about doing things that will embarrass myself or others, because of my eyesight	68.5%	10.9%	6.4%	5.8%	8.3%

Around 30.9% to 43.5% of the participants reported problems related to eyesight that was faced by them to some extent. The most commonly agreed upon problems were having much less control over what they do, because of their eyesight and feeling frustrated a lot of the time because of their eyesight, Table 3.

### Dry eye syndrome

Around one third the study participants (29.0%) reported that have been diagnosed by a clinician as having dry eye syndrome. A total of 15.3% of them reported that they feel their eyes are dry (not wet enough) and 17.3% reported that they feel their eyes are irritated, Table 4. Based on Women’s Health Study Questionnaire (WHS) criteria, a total of 479 participants (33.4%) are symptomatically diagnosed with DES.

**Table 4:** Dry eye syndrome symptoms assessment

Demographic variable	Overall (*n* = 1,431)	Iraq (*n* = 1,018)		Jordan (*n* = 413)
	Frequency (%)	Frequency (%)		Frequency (%)
1. Have you ever been diagnosed by a clinician as having dry eye syndrome?				
Yes	415 (29.0%)	247 (24.3%)		168 (40.7%)
Frequency	Constantly	Often	Sometimes	Never
2. How often do your eyes feel dry (not wet enough)?	39 (2.7%)	181 (12.6%)	631 (44.1%)	580 (40.5%)
3. How often do your eyes feel irritated?	23 (1.6%)	225 (15.7%)	810 (56.6%)	373 (26.1%)

### Risk factors of dry eye syndrome

Binary logistic regression analysis confirmed that students aged 27–29 years, those at their fifth year of study, and those who wear contact lenses are at higher risk of developing DYS compared to others with (Odds ratio (OR) 1.44 (95% CI: 1.00–2.08)), (OR: 2.11 (95% CI: 1.41–3.16)), and (OR: 1.70 (95% CI: 1.24–2.32)), respectively. On the other hand, students who are at their third year of study, those who reported that they have chronic diseases showed lower risk of developing DYS compared to others with (OR: 0.69 (95% CI: 0.51–0.94)) and (OR: 0.46 (95% CI: 0.31- 0.69)), respectively, Table 5.

**Table 5:** Binary logistic regression analysis

**Demographic variable**	**Odds ratio of having dry eye syndrome** (95% CI)
**Gender**	
Males (Reference group)	1.00
Females	0.87 (0.68–1.10)
**Age category**	
18–20 years (Reference group)	1.00
21–23 years	1.09 (0.87–1.37)
24–26 years	0.86 (0.65–1.13)
27–29 years	1.44 (1.00–2.08)*
30 years and above	1.31 (0.88–1.97)
**Field of study**	
Medical college (Reference group)	1.00
Non-medical college	0.84 (0.67- 1.04)
**Marital status**	
Single (Reference group)	1.00
Married	0.97 (0.73–1.28)
Divorced	1.54 (0.67–3.54)
Widowed	1.00 (0.09–11.00)
**Year of study**	
First year (Reference group)	1.00
Second year	0.90 (0.68–1.19)
Third year	0.69 (0.51–0.94)*
Fourth year	1.16 (0.89–1.51)
Fifth year	2.11 (1.41–3.16)***
Sixth year	1.06 (0.45–2.52)
Higher education	0.93 (0.68–1.25)
**Income level**	
Less than 700 USD (Reference group)	1.00
700 – 1500 USD	1.10 (0.84–1.43)
1500 – 2000 USD	1.09 (0.70–1.69)
More than 2000 USD	0.94 (0.53–1.67)
**Do you suffer from any chronic conditions?**	
No (Reference group)	1.00
Yes	0.46 (0.31- 0.69)***
**Contact lens user**	
No (Reference group)	1.00
Yes	1.70 (1.24–2.32)**
**Screen time** (mobile, television, laptop, etc.…) **(hours/day)**	
Less than 2 h (Reference group)	1.00
2–4 h	0.69 (0.47–1.00)
4–6 h	1.11 (0.86–1.42)
6–8 h	1.02 (0.80–1.31)
8 h and above	1.11 (0.89–1.41)

^*^p < 0.05, **p < 0.01, ***p < 0.001

## Discussion

The aim of this study was to explore the visual health and prevalence of dry eye syndrome among university students in Iraq and Jordan. The key findings are: 1) around one third the study participants are diagnosed symptomatically with DES, 2) a total of 15.3% of them reported that they feel their eyes are dry (not wet enough) and 17.3% reported that they feel their eyes are irritated, 3) more than half of them reported that their screen time is more than 6 h per day, 4) a total of 9.2% of them reported suffering from severe to very severe pain or discomfort in and around their eyes (for example, burning, itching, or aching), 5) around half of the study participants (50.8%) reported that at some point of the time they accomplish less than what they would like because of their vision, 6) students aged 27–29 years, those at their fifth year of study, and those who wear contact lenses are at higher risk of developing DES compared to others.

The majority of participants reported that they spent more than 6 h per day on their devices. One of the main contributing factors to DES is long screen time. In one Egyptian study, they found that information technology (IT) professionals who worked more than 6 h per day on their computers started to have symptoms of DES as 80% of them felt headaches, 70% blurred vision, and 76% burned eyes [29]. One of the reasons is that when people are using different devices, they focus on the screen for long periods without even blinking their eyes [3]. Unfortunately, during the COVID-19 pandemic isolation, many people of all different ages started to use digital devices daily for more than 5 h per day [19]. In one Saudi Arabian study, researchers compared the hours of device use before and after curfew and compared the results on digital eye strains. The results indicated that the DES increased by 76% during the curfew [30].

Approximately one-quarter of the study participants evaluated their overall health as good or excellent. However, around 41% of them did not report good eyesight. It is highly important for all people who have been using electronic devices for a long time to check their eyesight regularly. They have to make sure that they are using the optimal prescription for their eyes to minimize their visual eye strain. Improper visual sight will affect their quality of life and their productivity. Irritation, burning sensations, redness, tearing, pain, and blurred vision are symptoms that cause people to be absent or reduce their focus and productivity [8].

About 16% of people said they are concerned about their eyes most of the time, if not all of the time. Most of the participants reported that they had much less control over what they were doing and felt frustrated most of the time because of their eyesight. Worrying is a normal emotion that occurs as a result of DES symptoms. In one previous study, they compared the relationship between DES and mental health. The results of the study found that there was a direct correlation between DES's poorer health status and self-psychological stress burden. As a result, it is critical to seek medical attention to minimize DES symptoms, as this may increase the patient's anxiety [31].

Around 10% of the participants reported having severe to very severe symptoms around their eyes. One Saudi Arabian study found that the decrease in work productivity was higher among people who were suffering from severe symptoms. The results also showed that these people not only decreased their performance at work but also at home, which affected their overall quality of life [32]. In our study, the most commonly reported activities that participants struggled with reading street signs or the names of stores, reading ordinary print in newspapers, and finding something on a crowded shelf. Around half of the study participants reported that they accomplish less than what they would like to do because of their vision. DES resulted in difficulty focusing on things and increased braking time during work. We rely on electronic devices more to accomplish different tasks. Most of the time, workers use their laptops, I-pads, and smartphones to send emails, join virtual meetings, and accomplish their work goals. Moreover, university students rely on electronic devices to communicate with their colleagues and professors, do research and expand their knowledge.

In around one-third of the study, participants reported having been diagnosed with DES by a clinician. Some of them reported dry eyes and irritating eyes. To diagnose DES, doctors do a comprehensive examination of patient symptoms, their medical history, and their previous tropical or systematic medication, and ask the patients if they have been exposed to many factors that cause irritation to their eyes, to enable them to diagnose the cause of DES. After that, doctors do a diagnostic test and an ophthalmological examination. Some tests are used to examine the tear and others for ocular surface integrity. To examine the quality of tears, there is a tear film break up time test (TBUT), and Schirmer testing is used to examine the volume of tears. More tests, such as the tear film osmolarity test to measure the particles and water within the tears, and MMP-9 testing [21, 33]. To examine the ocular surface integrity, doctors can use different stains and dyes, such as fluorescein stain and Lissamine green dye [21].

There are different questionnaires for the diagnosis of DES, such as the Dry Eye Questionnaire, the McMonnies Questionnaire, and the Ocular Surface Disease Index (OSDI) questionnaire. This questionnaire-based on 12 questions with scores from 0 to 100. The higher the score of the patient, the more severe his case is [32, 34]. The Symptom Assessment iN Dry Eye (SANDE). The SANDE questionnaire asks the patient about the frequency and severity of their symptoms [33]. The Rasch-based Computer-Vision Symptom Scale (CVSS17) and the self-administered Computer Vision Syndrome Questionnaire (CVS-Q), both are used to assess DES among computer users [8]. National Eye Institute Visual Functioning Questionnaire – 25 (VFQ-25), a 25-item questionnaire used to assess eye health and visual problems [35]. The Women’s Health Study Questionnaire (WHS) to determine if there was a previous diagnosis of DES and the severity of the disease [25]. The results of this study were similar to the WHS study, with confirmed diagnosis percentages of 29.0% and 33.4%, respectively.

Students in their fifth year of study, and those who wear contact lenses are at a higher risk of developing DES than others. The possible reason for that is that fifth-year students have a graduation project that they need to spend a lot of time using their electronic devices to do, which will increase their risk of developing DES. Moreover, wearing contact lenses will divide the tear film into two layers, which will cause instability, decrease the thickness of the tear film layer and increase the friction between the ocular surface and the contact lens [36].

To decrease the prevalence of DES, the government and policymakers should increase public awareness of the importance of taking care of the eyes. A possible suggestion is an awareness campaign. Also, universities should increase awareness among their students. It is good to mention the 20–20-20 rule to minimize their eye strain while using electronic devices, such as every 20 min working on the screen, students should look for something 20 feet away for 20 s.

This study has multiple strengths. This study was one the few and the largest (1,431 university students) studies in the Arab world and the Middle East to look at visual health and the prevalence of dry eye syndrome among university students. At the same time, this study has limitations. Since we distributed the questionnaire online link over various media platforms (WhatsApp, Facebook, and Snapchat) to reach a large number of participants, we were unable to estimate the number of universities from which our study participants came. Our ability to determine causality between research variables was limited by the study design, which was a cross-sectional survey. Finally, we were unable to determine the response rate for our study, which may have resulted in nonresponse bias because we were unable to demonstrate how well the sample was chosen from the target group. We did not estimate the average temperature or humidity during the study period, and we believe the results would have been different if the study had been conducted during the summer. As a result, the findings must be interpreted carefully.

## Conclusion

Dry eye syndrome is common health problem among university students. The time university students spend working with video display terminals increases in the synchronous hybrid learning environment, as does the prevalence of dry eye complaints. It should not be overlooked that dry eye might have a negative impact on academic performance and their overall quality of life. Further studies are required to identify other risk factors associated with DES. Future research should focus on identifying strategies that could help reduce the risk of developing DES as a result of the inevitability of long-term use of digital devices among many categories of society, including university students.

## Data Availability

The datasets used and/or analysed during the current study are available from the corresponding author on reasonable request.
